# Editorial: Neurodegeneration: From Disease Mechanism to Therapeutic Advancement

**DOI:** 10.3389/fgene.2022.941846

**Published:** 2022-06-14

**Authors:** Partha S. Sarkar, Giovanni Meola, Nan Zhang

**Affiliations:** ^1^ Department of Neurology, University of Texas Medical Branch, Galveston, TX, United States; ^2^ Department of Neurorehabilitation Sciences, Casa di Cura del Policlinico, University of Milan, Milan, Italy; ^3^ Department of Neurology, Neuroscience Program, Houston Methodist Research Institute, Houston, TX, United States

**Keywords:** neurodegeneration, alzheimer’s disease, parkinson’s disease, amyloid, alpha-Synuclein, single-cell RNA sequencing, nanoparticle, dm1

Neurodegenerative diseases selectively affect vulnerable brain regions, cause progressive mental and physical debilitation, and collectively cost over $655 billion a year in the US. Although many pre-clinical and clinical trials are currently underway with groundbreaking technologies such as CRISPR and oligonucleotide therapies, most neurodegenerative diseases remain incurable, partly due to the lack of a precise understanding of disease mechanisms, and partly due to the lack of appropriate disease models that can fully recapitulate human disease phenotypes. With these in mind, we collected two reviews and six original research articles in the hope of shedding light on new pathomechanisms of neurodegeneration, therapeutic innovation and biosafety assessment.

Alzheimer’s disease (AD) and Parkinson’s disease (PD) are the most common neurodegenerative diseases worldwide; both affect neuronal function, movement and cognition. Jiang et al. reviewed the amyloid β (Aβ) cascade with respect to three topological forms in AD pathogenesis, the neurotoxicity arising from spontaneously aggregated α-synuclein fibrils in PD, and the receptor-mediated cell-to-cell transmission mechanisms of the prion-like misfolded proteins. The authors also reviewed the recent development and applications of single-cell RNA sequencing (scRNA-seq) in AD and PD. Given the technical limitations of traditional scRNA-seq, such as unfavorable brain dissociation treatment and inability to resolve spatial and positional patterns, the authors suggested that single nuclear RNA-seq (snRNA-seq) may be an alternative strategy that is better suited for frozen clinical samples with a higher cell subtype resolution.

Silicon nanoparticles (SiO_2_ NPs) are extensively used for drug delivery, molecular imaging and gene therapy, owing to their high dispersibility, stability and biocompatibility. However, Yuan et al. demonstrated that SiO_2_ NPs triggered α-synuclein deposition, mitochondrial impairment and autophagic inhibition *in vitro* as well as enhanced dopaminergic neuronal death in PD transgenic mice post intranasal administration. These observations are in line with SiO_2_ NPs’ roles in air pollution, neurotoxicity via the gut-brain axis, and promotion of AD pathology. The therapeutic applications of SiO_2_ NPs may warrant caution.

The familial form of PD represents 5–10% cases with either dominant or recessive inheritance. Although many causal genes and mutations have been identified, the genetic predisposition of many PD patients are still unknown. Kolarikova et al. performed whole exome sequencing on PD subfamily trios of the southeastern Moravia population; this Czech republican population is known to have a higher-than-average prevalence of PD. The authors identified six rare genetic variants that could contribute to PD. These mutated genes play diverse cellular roles, including pigmentation, microtubule function, viral transcription, endocytosis, immune response and vesicle trafficking.

Astrocytes are the most abundant glial cells in the brain and they safe-guard neurons by providing nutrients, remodeling synapses, regulating ion homeostasis, and forming the blood-brain barrier (BBB). Both astrocytic senescence and astrogliosis surrounding Aβ plaques have been observed in AD, but the underlying pathological mechanisms remain unclear. Deng et al. cultured control and Aβ-treated primary astrocytes for 90 days and performed RNA-seq analysis. By comparing differentially expressed long non-coding RNA, circular RNA, microRNA and messenger RNA, the authors found prominent changes in the focal adhesion signaling pathway, the extracellular matrix receptor signaling pathway and the extracellular matrix in Aβ-treatment senescent astrocytes. This study may help uncover new targets arisen from astrocytic toxicity in AD, in addition to those from the current neuron-centric disease cascade.

In a parallel investigation, Hasuike et al. studied senescence of primary human lung fibroblasts that conditionally express an expanded CUG repeat. This cell line was used to model myotonic dystrophy type 1 (DM1)—a multisystemic neurodegenerative disease caused by CUG expansion in the *DMPK* (*DM1 protein kinase*) gene. The authors found that the expression of expanded CUG repeats in cells induced premature senescence, which is supported by biomarker studies, RNA-seq analysis and cell cycle checkpoint inhibitor activation. The expression of pathologically expanded CUG repeats also caused DNA damage repair activation, mitochondrial dysfunction, and ROS (reactive oxygen species) production.

Epilepsy affects 1–2% of the world’s population and represent a clinical feature of neurodegeneration. Although the upregulation of nuclear factor kappa B (NF-κB) is well established in patient and animal brains with epilepsy, the complex role of NF-κB is not fully understood. Cai et al. reviewed the spatiotemporal expression of NF-κB in epileptic brains and the impact of its dysregulation on neuronal survival, inflammation, oxidative stress, glial activity and BBB permeability. The authors highlighted the neuroprotective role of NF-κB as well as its damaging effect on the central nervous system (CNS) by inducing inflammatory response and antioxidant imbalance during epilepsy.

Progranulin (PGRN) critically supports the health of the CNS, and its targeted expression slows the onset of neurodegeneration. By creating stable PGRN-depleted and -overexpressing NSC-34 motor neuron cells and by employing a parallel omics approach, Chitramuthu et al. established the roles of PGRN in cytoskeleton and synaptic differentiation, neurotrophic receptor homeostasis, and endoplasmic reticulum/Golgi apparatus/lysosome regulation.

Diabetic retinopathy (DR) is a leading cause of vision loss worldwide and the most common microvascular complication of diabetes. The rate of retinal neurodegeneration varies greatly between individuals. By assessing the retinal layer thickness in type 2 diabetic patients over a 5-year period, Madeira et al. observed significant retinal neurodegeneration across all patient groups but distinct retinopathy profiles. These phenotypic distinctions may offer personalized management of DR.

To summarize, our special issue covers a breadth of topics, including prion-like protein aggregation and cell transmission, state-of-the-art omics technologies, nanoparticle safety profiles, cell senescence and longitudinal patient studies across multiple neurodegenerative diseases by using computational, human, animal and cell models. We hope that our special issue will stimulate further discussion and help develop novel disease interventions that could improve patients’ quality of life.

